# Clinically Evident Portal Hypertension Is an Independent Risk Factor of Hepatocellular Carcinoma Recurrence Following Liver Transplantation

**DOI:** 10.3390/jcm14062032

**Published:** 2025-03-17

**Authors:** Arno Kornberg, Nick Seyfried, Helmut Friess

**Affiliations:** Department of Surgery, Klinikum Rechts der Isar, Technical University of Munich (TUM), Ismaninger Str. 22, 81675 Munich, Germany; nick.seyfried@tum.de (N.S.); helmut.friess@tum.de (H.F.)

**Keywords:** clinically evident hypertension, hepatocellular carcinoma, liver transplantation, tumor recurrence, transarterial chemoembolization, ^18^F-fluorodeoxy-glucose positron emission tomography, inflammation

## Abstract

**Background/Objectives**: Clinically evident portal hypertension (CEPH) is a major risk factor for the development and poor outcomes of hepatocellular carcinoma (HCC). The aim of this study was to determine the impact of CEPH on the risk of HCC recurrence following liver transplantation (LT). **Methods**: A total of 129 HCC patients were included in this retrospective analysis. The definition of CEPH was based on indirect clinical features without hepatic venous pressure gradient measurement. The impact of CEPH on the post-LT risk of HCC recurrence was determined by uni- and multivariate analysis. **Results:** Evidence of manifest portal hypertension (PH) was associated with a higher ^18^F-fluorodeoxy-glucose (FDG) uptake of HCC on positron emission tomography (PET; *p* < 0.001) and increased serum levels of C-reactive protein (*p* = 0.008) and interleukin−6 (IL-6; *p* = 0.001). The cumulative risk of HCC recurrence at 5 years post-LT was significantly higher in the CEPH group (38.1% vs. 10.6%, *p* < 0.001). The eligibility for neoadjuvant transarterial chemoembolization (TACE) was comparable between both study cohorts (71.4% vs. 74.2%; *p* = 0.719). However, the post-interventional pathologic response rate was significantly lower in the case of PH (15.6% vs. 53.1%; *p* < 0.001). In addition to the Milan criteria (MC), ^18^F-FDG avidity on PET and serum values of IL-6 and alfa-fetoprotein, we identified CEPH as another significant and independent predictor of HCC recurrence (*p* = 0.008). **Conclusions**: CEPH correlates with an unfavorable tumor phenotype, TACE refractoriness and a risk of post-LT HCC recurrence. Therefore, the clinical features of PH should be implemented in pre-transplant risk assessment and decision-making processes.

## 1. Introduction

Liver transplantation (LT) is widely recognized to provide the best long-term prognosis in patients with early-stage hepatocellular carcinoma in liver cirrhosis. By strictly adhering to upper tumor burden limits as defined by the most popular Milan criteria (MC), post-LT recurrence-free survival probabilities beyond 70% may be achieved, which are quite comparable to those reported in non-malignant transplant indications [1]. However, LT prioritization exclusively determined by tumor size and number proved to be too rigid to meet complex requirements that are arising from a rapidly changing LT landscape in times of an aggravating donor organ shortage. Clearly, discriminatory capacities of conventional LT selection models may be substantially improved by incorporating clinical surrogates of tumor biology, such as serum alfa-fetoprotein (AFP) [2], responses to locoregional treatments [3] and metabolic uptake patterns on ^18^F-fluorodeoxy-glucose (FDG) positron emission tomography (PET) [4].

There is a growing body of evidence that, in addition to intrinsic tumor aggressiveness, the characteristics of the liver recipient, such as background liver disease and functional performance, have a significant influence on both tumor recurrence risk and overall survival (OS) [5,6]. Progressive fibrotic/cirrhotic liver remodeling has the potential to elevate vascular resistance and portal vein pressure (PP), which may finally result in manifest portal hypertension (PH). Apart from the opening of pre-existing collateral vessels and porto-systemic shunts, the mechanisms of neo-angiogenesis triggered by the upregulation of various pro-inflammatory and -angiogenic cytokines and growth factors were recently demonstrated to play a major role not only in hepatocarcinogenesis but also in the occurrence and severity of PH [7]. While transjugular hepatic venous pressure gradient measurement (HVPG) ≥ 10 mmHg still represents the gold standard for the diagnosis of clinically evident portal hypertension (CEPH), non-invasively assessed surrogates like esophago-gastric varices, ascites and splenomegaly are generally used in clinical routines for patient evaluation [8].

Undoubtedly, CEPH is closely associated with the development, progression and treatment response of HCC [7,9]. Despite significant advancements in patient selection and surgical techniques, including minimally invasive approaches, PH is still a major risk factor for liver decompensation and early tumor relapse following hepatectomy and non-surgical interventions [10]. Therefore, LT is considered to be the most feasible therapeutic option for achieving both curation from liver malignancy and the reversal of porto-systemic perfusion disturbances. Nonetheless, the oncologic impact of CEPH in the setting of LT remained, so far, undefined. This study aimed to determine the predictive value of CEPH based on indirect clinical features in cirrhotic patients with specific regard to HCC recurrence following LT.

## 2. Materials and Methods

### 2.1. Study Cohort

A total of 129 HCC patients who underwent LT between 2000 and 2015 were identified in a prospectively managed LT database and included in a retrospective analysis. The study was conducted in compliance with the guidelines of the Declaration of Helsinki. The Institutional Review Board of the ethical committee of the Technical University Munich has approved the study (Nr. 217/15). Diagnosis of HCC was based on cross-sectional imaging without performing tumor nodule biopsy [11]. LT prioritization was based on waiting time and Child–Pugh score until December 2007 and on the model of end-stage liver disease (MELD) score thereafter. The MC represented the tumor-specific standard for LT registration (Milan-in). Depending on a positive interdisciplinary transplant board vote, patients with HCC that barely did not meet or exceed the MC during waiting time (Milan-out) could also be considered for LT without, however, being managed with MELD priorities. Patients with rapid multifocal tumor progression, macrovascular invasion, extrahepatic spread and tumor-related symptoms were excluded from LT.

All registered patients underwent ^18^F-FDG PET/computed tomography (CT) at least once to refine tumor staging. Ratio of tumor-to-normal liver standardized uptake value (T_SUVmax_/L_SUVmax_) was assessed to describe metabolic tumor aggressiveness. Patients were classified as PET-positive (T_SUVmax_/L_SUVmax_ > 1, ^18^F-FDG-avid) or PET-negative (T_SUVmax_/L_SUVmax_ = 1, ^18^F-FDG-non-avid), as previously described [4,12]. Pre-transplant serum levels of C-reactive protein (CRP) and interleukin−6 (IL-6) were determined for assessment of systemic inflammatory immune response [13].

The indication for super-selective transarterial chemoembolization (TACE) as neoadjuvant bridging concept was discussed interdisciplinarily by considering functional hepatic capacity and tumor burden. Recent episodes of hepatic decompensations within 3 months, Child–Pugh C cirrhosis, serum bilirubin > 3 mg/dL and irregular branches of the hepatic artery were considered as basic contraindications for neoadjuvant TACE. Post-interventional tumor necrosis rate of > versus ≤75%, as determined by explant liver pathology, indicated response versus non-response to TACE [14].

### 2.2. Definition and Assessment of CEPH

As previously described, presence of one or more of the following indirect clinical signs was applied to define CEPH: (1) ascites; (2) esophago-gastric varices; (3) splenomegaly (>12 cm in the axial plan) + thrombocytopenia (<100,000 G/L) [7,8,10]. Data from tomographic imaging and esophagoscopy were used for evaluation of splenomegaly and esophago-gastric varices. For the assessment of CEPH severity, the following grading system based on number of defining clinical features was used: grade 0: none (no CEPH); grade 1: up to 2 features; grade 3: 3 features [15].

### 2.3. Transplant Procedure and Post-LT Surveillance

Standard deceased donor LT was performed in all study patients without application of veno-venous bypass. Hepatic venous reconstruction was realized by using the piggy-back technique, and bile duct reconstruction was performed by end-to-end or biliodigestive anastomosis without insertion of a T-tube [16]. Besides tumor load, histopathologic analysis of the explanted livers focused on grading and microvascular invasion (MVI) as established features of intrinsic tumor biology. A calcineurin inhibitor-based dual regimen consisting of either cyclosporine A or tacrolimus in combination with mycophenolate mofetil was implemented for maintenance immunosuppressive therapy. All liver allograft recipients underwent a standardized post-transplant surveillance protocol consisting of liver ultrasound and serum AFP level measurement every 3 months and cross-sectional imaging of the chest and abdomen at least twice a year. Percutaneous biopsy and/or ^18^F-FDG PET/CT were additionally performed, if necessary, for further evaluation.

### 2.4. Outcome Variables

In addition to CEPH and defining features, the following available pre-transplant clinical variables were included in our risk analysis: 1. Basic data: sex, age, etiology of cirrhosis; 2. Liver function: MELD score and Child–Pugh score; 3. Tumor characteristics: number and total diameter of tumor nodules, MC, serum level of AFP (normal value ≤ 6 ng/mL), TACE, ^18^F-FDG uptake on PET. 4. Systemic inflammatory response: serum level of CRP (normal value < 0.5 mg/dL) and IL-6 (normal value < 7 ng/L).

Post-LT assessed histopathologic data were not included in the analysis since these features were not available prior LR and may, therefore, not be applied for individual risk assessment.

### 2.5. Statistical Analysis

All statistical analyses were performed using SPSS 25.0 software (IBM Inc., Munich, Germany). Data are expressed as median and range or mean ± standard deviation (STD) and compared using Mann–Whitney U test or *t*-test. Chi square test or Fisher’s test was used to analyze categorical variables. Post-transplant rates of cumulative HCC recurrence and OS were calculated using the Kaplan–Meier method and compared using log-rank test. A logistic regression model was applied to identify independent predictors of post-LT HCC recurrence. A *p* value < 0.05 was considered statistically significant.

## 3. Results

### 3.1. Clinicopathologic Characteristics

The characteristics of the study cohort are demonstrated in Table 1.

Sixty-three patients (48.8%) suffered from CEPH before LT. Diagnosis was based on one, two or three defining features in 24 (38.1%), 27 (42.9%) and 12 LT candidates (19%). Accordingly, CEPH was classified grade 0 in 66 patients (51.2%), grade 1 in 51 patients (39.5%) and grade 2 in 12 patients (9.3%), respectively. Evidence of PH was associated with a higher MELD and Child–Pugh score. Preoperative serum levels of CRP and IL-6 were both significantly higher in the case of PH, while no significant differences in the AFP level could be found. With regard to PET evaluation, CEPH correlated with significantly higher T_SUVmax_/L_SUVmax_ values and overall ^18^F-FDG avidity rates. Tumor size characteristics, including the MC, were not different. Explant liver histopathologic analysis demonstrated higher rates of poor tumor differentiation and MVI in the PH group (Table 1).

### 3.2. Post-LT Outcome and Risk Factors of HCC Recurrence

**Figure 1:**
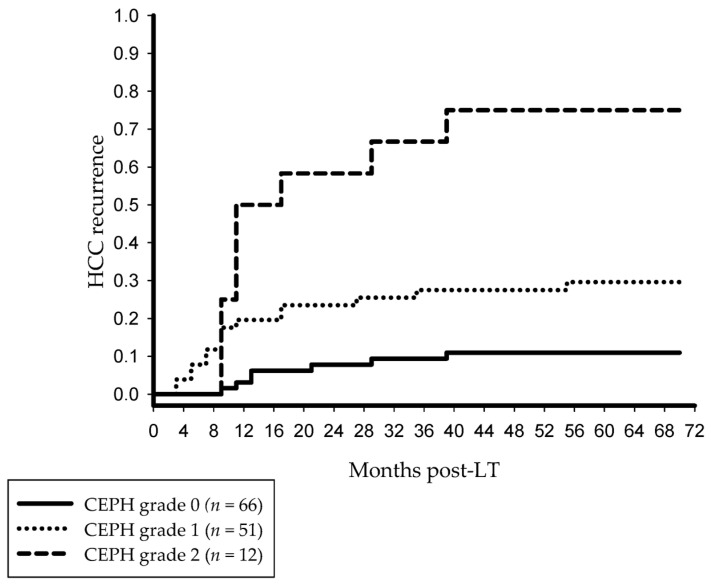
Cumulative HCC recurrence rates at 3 and 5 years post-LT were 9.4% and 11% without PH (grade 0), 27.5% and 29.6% in grade 1 CEPH (*p* = 0.009) and 66.7% and 75% in grade 2 CEPH (*p* = 0.006 vs. grade 1; *p* < 0.001 vs. grade 0), respectively. CEPH, clinically evident portal hypertension; HCC, hepatocellular carcinoma; LT, liver transplantation.

Post-transplant OS rates at 3 and 5 years tended to be better without PH (91.8%, 79% versus 75%, 60%; *p* = 0.314). The overall HCC recurrence rates were 38.1% and 10.6% with and without CEPH (*p* < 0.001). Relapse probabilities were 29.2%, 29.7% and 75%, respectively, in case of one, two and three defining features (*p* < 0.001). Oncologic risk stratification according to the grading of CEPH is illustrated in Figure 1.

**Figure 2:**
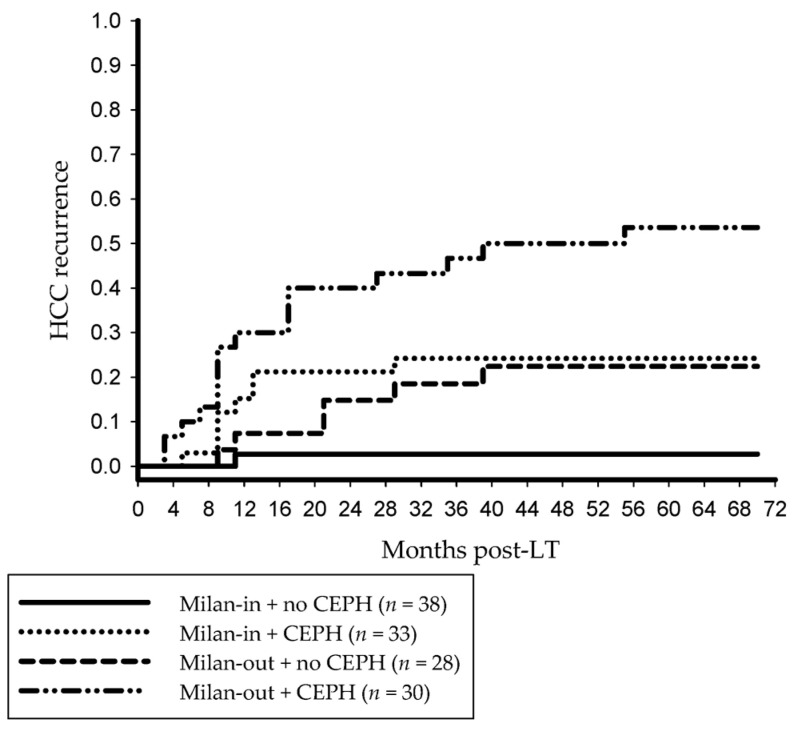
Evidence of PH was a significant oncologic risk factor for both Milan-in (*p* = 0.007) and Milan-out patients (*p* = 0.013). Cumulative HCC recurrence risk in Milan-out patients without PH (22.4%) was comparable to both portal hypertensive Milan-in patients (24.2%; *p =* 0.766) and the overall Milan-in group (12.8%; *p* = 0.287). CEPH, clinically evident portal hypertension; HCC, hepatocellular carcinoma; LT, liver transplantation.

Evidence of PH was associated with a significantly higher tumor recurrence risk in both patients meeting and those exceeding the MC. Tumor recurrence rates were comparable between Milan-out patients without CEPH and Milan-in recipients (Figure 2).

Along with Milan-out status, ^18^F-FDG avidity and elevated serum levels of IL-6 and AFP, CEPH could be identified as another significant and independent predictor of HCC recurrence (Table 2). CEPH remained an independent prognostic factor after the inclusion of MVI and grading in multivariable analysis.

### 3.3. CEPH and TACE

**Figure 3:**
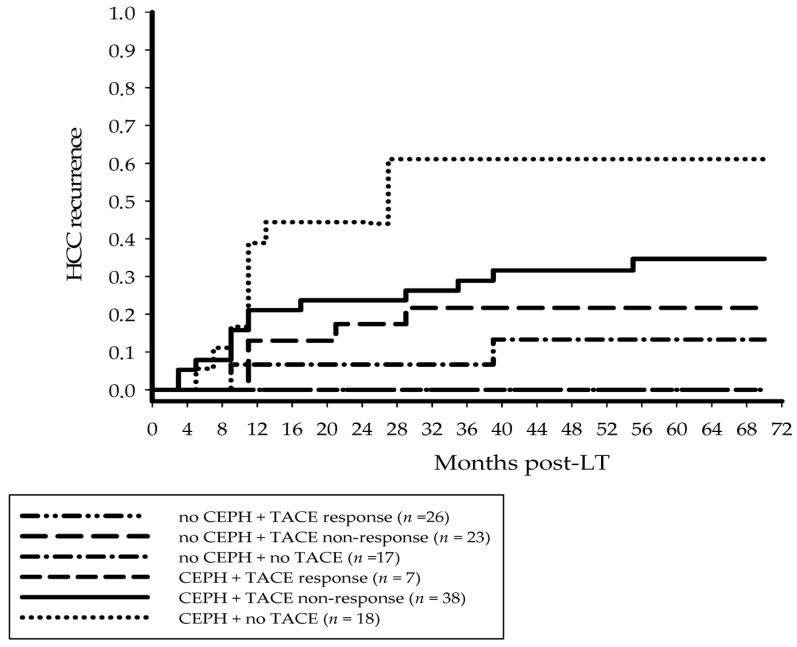
In the non-CEPH subset, cumulative HCC relapse rates at 3 and 5 years post-LT were 0% and 0% in case of TACE-response, 21.7% and 21.7% in TACE non-response (*p* = 0.013) and 6.7% and 13.3% without neoadjuvant treatment (*p* = 0.061 versus responders; *p* = 0.523 versus non-responders). Corresponding recurrence probabilities in the CEPH group were 0% and 0% in responders, 28.9% and 34.7% in non-responders (*p* = 0.086) and 61.1% and 61.1% without TACE (*p* = 0.012 versus responders; *p* = 0.039 versus non-responders). CEPH, clinically evident portal hypertension; HCC, hepatocellular carcinoma; LT, liver transplantation; TACE, transarterial chemoembolization.

Tumor recurrence rates were 19.1% and 37.1% in patients with and without neoadjuvant TACE (*p* = 0.033). In detail, recurrence risk at 5 years post-LT was 0%, 29.7% and 39.3%, respectively, in TACE responders, non-responders to TACE and patients who did not receive neoadjuvant therapy (*p* = 0.001 response versus non-response, versus no TACE; *p* = 0.293 non-response versus no TACE). TACE applicability was comparable between both study cohorts (74.2% versus 71.4%; *p* = 0.719). There was no case of severe post-TACE hepatic decompensation. However, the pathologic TACE response rate was significantly lower in portal hypertensive liver recipients (15.6% versus 53.1%; *p* < 0.001). The oncologic risk in PH patients proved to be significantly lower in TACE non-response compared to non-pretreatment, while being comparable between both corresponding subsets without CEPH (Figure 3).

## 4. Discussion

To the best of our knowledge, this is the first study to demonstrate a significantly higher risk of HCC recurrence in liver transplant patients suffering from CEPH. Along with the predictive determinants of tumor burden (MC), biological tumor aggressiveness (AFP level, ^18^F-FDG avidity) and systemic inflammatory state (IL-6 level), CEPH could be identified as another significant and independent predictive factor in our multivariable analysis. There is convincing evidence on the prognostic importance of background liver disease and hepatic functional reserve in HCC patients even following replacement of the cirrhotic tumor liver by an adequately functioning hepatic allograft [5,6,17]. Consequently, the incorporation of established liver function parameters like MELD score and Child–Pugh classification has been recommended to refine risk stratification and patient selection [18]. Indeed, both of them proved to be strong prognostic factors in our LT series without, however, achieving independent predictive value in the interplay with CEPH. Moreover, our data suggested a collinearity between the severity of PH and post-transplant oncologic risk. Thus, pending verification in a larger study approach, features of portal venous hemodynamic disturbances rather than parameters of liver function should be applied to assess the cancerogenic impacts related to cirrhotic alterations. In fact, a previous French study was able to identify CEPH as an independent risk factor of tumor-related waiting list dropout and OS in a series of 243 consecutively listed LT candidates but without identifying a specific impact on post-transplant HCC recurrence [19].

Clearly, PH is predictive for the development and prognosis of HCC independently from underlying liver damage, whilst tumors may increase PP by changing liver architecture and/or infiltrating into hepatic vessels [20,21]. Interestingly, there were no significant differences regarding tumor size criteria between our study groups. Of note, no significant outcome difference could be found between Milan-out patients without PH and Milan-in patients, whereas more than 50% of portal hypertensive MC-out patients have so far developed HCC relapse (Figure 2). This finding implicated clinical features of PH to be helpful for identifying advanced HCC patients with low-aggressive phenotype that might benefit from LT despite exceeding current standards of acceptable tumor burden limits. This could be particularly interesting for LT centers that are following a liberal section policy and should, therefore, be further evaluated.

Significantly higher ^18^F-FDG uptake on pre-transplant PET of portal hypertensive patients supported the idea of mechanobiological processes to foster aggressive tumor behavior and the risk of post-transplant recurrence. Although diagnostic accuracy is considered insufficient to qualify as a standard method in HCC imaging, ^18^F-FDG PET/CT may be very valuable for accurate tumor staging, predicting treatment response and the detection of recurrence. In addition, ^18^F-FDG avidity was shown to be highly predictive for poor differentiation and MVI, both of which are regarded as crucial risk factors for poor survival [4,12,22]. Indeed, evidence of PH was associated with a higher risk of unfavorable histopathologic phenotype in our study. However, besides correlating with intrinsic tumor aggressiveness, enhanced ^18^F-FDG uptake may also be related to inflammation and hypoxia, which are fundamental neo-angiogenic stimuli in both progressive liver cirrhosis and hepatocarcinogenesis [22,23]. Pro-inflammatory mechanisms in the context of background cirrhosis worsen oxygen supply to hepatocytes, foster vessel permeability and promote recruitment of different leucocytes that are releasing a wide range of pro-angiogenetic cytokines, such as vascular endothelial growth factor (VEGF). The resulting neovessels are, however, structurally and functionally of immature nature, leading to high vessel porosity, interstitial edema, hypoxia and necrosis [24]. By stimulating myeloid-derived suppressor cells, regulatory T-cells and tumor-associated macrophages, VEGF was also shown to mediate an immunosuppressive tumor microenvironment (TME) and, thereby, HCC progression and metastasis [25]. Significantly higher T_SUVmax_/L_SUVmax_ values could be an indication of a more “aggressive” TME in patients with CEPH, as ^18^F-FDG uptake was recently shown to also correlate with a number of infiltrating cytotoxic and inflammatory immune cells [26]. Pre-transplant higher serum levels of CRP and IL-6 observed in our CEPH group seem to support this hypothesis, as they may not only reflect systemic inflammation but were also shown to correlate with immune status at the tumor site [27,28]. Notably, elevated IL-6 levels even proved to be an independent predictive factor in our analysis, which underlined the prognostic importance of inflammatory immune response in HCC recurrence.

Nonresolving inflammation may also have contributed to the higher rate of TACE refractoriness observed in the CEPH cohort [29]. Post-interventional pathologic response rates were 53.1% with but only 15.6% without evidence of PH. This was another result with prognostic significance in our LT series, since response to TACE was associated with a 0% HCC recurrence risk and, thus, presumably with an oncologic cure. In light of growing insight into the complex interplay between immunogenic TME, systemic inflammation and treatment response, we suppose the infiltration of immunosuppressive cell populations combined with hypoxic extracellular remodeling to create a state of immune escape, rendering tumors in portal hypertensive cirrhotics more resistant against neoadjuvant TACE [30,31]. In line with this, we were able to identify a close link between the ^18^F-FDG avidity of HCC, TACE refractoriness and poor outcome in a previous LT study [14]. Others reported on elevated VEGF levels to correlate with poor TACE results, while adding anti-angiogenic medication by sorafenib improved post-interventional outcomes [31].

Generally, TACE should be indicated with caution in PH since it may be associated with an increased risk of hepatic decompensation, tumor progress and poor survival [15]. But, in agreement with the study by Faitot et al. [19], we did not find a significant difference in TACE eligibility between our study groups. Besides the application of a super-selective TACE approach, the exclusion of recently decompensated LT candidates may have significantly contributed in controlling post-TACE decompensation risk in our CEPH subset, as the Child–Pugh score alone was shown to be inappropriate to adequately correlate with re-compensated liver function. The implementation of additional useful tests to assess functional hepatic reserve, such as albumin–bilirubin score, indocyanine green clearance and specific nuclear medical diagnostics (e.g., 99 mTc-dimethyl-acetanilide-iminodiacetic acid scintigraphy), should probably be considered in this context not only for improving individual risk stratification but also to avoid unjustified refusal of neoadjuvant bridging treatments [32]. Despite a low pathologic response rate, TACE seems to provide an oncologic benefit in CEPH patients, as tumor recurrence rates were 28.9% with and 61.1% without neoadjuvant treatment (Figure 3). Thus, independent from achieving (near) complete tumor necrosis, increasing TACE amenability might be crucial for outcome improvement in this oncologic high-risk population. Transjugular intrahepatic porto-systemic shunting could play an important neoadjuvant role in this context since, contrary to earlier fears, it was recently shown to increase access to locoregional treatments without aggravating the risk of tumor cell spread [33]. In addition, there is growing evidence that the depressurization of the portal vein may not only prevent PH-related complications [34] but could also reduce the cancerogenic potency of a cirrhotic liver [35].

Given their capability to promote anti-tumor responses through the activation of infiltrating effector T cells, the implementation of immune checkpoint inhibitors is discussed to provide synergistic efficacies regarding TACE-induced immunogenic TME. Whether this will be an effective and tolerable neoadjuvant concept, specifically in patients suffering from PH, remains to be determined [36]. However, the adjustment of immunosuppressive treatment could be an already implementable tumor-suppressive concept in liver recipients with PH since an early reduction in CNI trough levels was shown to significantly reduce HCC recurrence risk [37]. The introduction of a mammalian target of rapamycin (mTOR) inhibitor like sirolimus may contribute to a safe CNI dose and level reduction without compromising the immunologic balance. Beyond that, mTOR inhibitors were shown to provide anti-angiogenic efficacies that could be specifically valuable with respect to PH-related neo-vascularization [38].

Besides its retrospective character and the relatively small subgroups, the lack of waiting list data, including drop-out features, is another limitation of our study, which made it impossible to conduct an intent-to-treat analysis. Moreover, investigations on a molecular level are required to obtain a more specific insight into the supposed association between PH, immunogenic TME and HCC recurrence risk. And finally, the exact prognostic importance of CEPH in our LT series remained, to some extent, speculative, since we did not routinely perform HVPG determination prior LT. There could be a differential predictive impact between invasively determined PP and indirect clinical signs of PH [39]. However, the application of clinical surrogates has the advantage of an immediate, safe and cost-effective evaluation of functional performance and decompensation risk. Against this background, further efforts should be made to identify highly predictive non-invasive tests of PH [40].

## 5. Conclusions

In conclusion, the findings of our study suggest CEPH to be an important risk factor of HCC recurrence following LT. Probably triggered by an immunosuppressive TME and systemic inflammation, evidence of PH is associated with lower TACE efficacy and, thereby, with inferior post-LT outcomes. Clinical features of CEPH could serve as surrogate markers of biological tumor aggressiveness and should, therefore, be implemented in pre-transplant risk stratification and waiting list management.

## Figures and Tables

**Table 1:** Clinicopathologic characteristics of the study cohort.

Prognostic Factors	All Patients (*n* = 129)	Without CEPH (*n* = 66)	With CEPH (*n* = 63)	*p* Value
Median age in years (range)	58.3 (38–71)	60.5 (38–71)	58.0 (41–70)	0.441
Sex, *n* (%) Female Male	53 (41.1) 76 (58.9)	28 (42.4) 38 (57.6)	25 (39.7) 38 (60.3)	0.752
Alcohol-related cirrhosis, *n* (%) No Yes	59 (45.7) 70 (54.3)	29 (43.9) 37 (56.1)	30 (47.6) 33 (52.4)	0.675
Viral cirrhosis, *n* (%) No Yes	87 (67.4) 42 (32.6)	49 (74.2) 17 (25.8)	38 (60.3) 25 (39.7)	0.092
Child–Pugh, *n* (%) A B or C	53 (41.1) 76 (58.9)	46 (69.7) 20 (30.3)	7 (11.1) 56 (88.9)	< 0.001
MELD score, *n* (%) ≤20 >20	72 (55.8) 57 (44.2)	56 (84.8) 10 (15.2)	16 (25.4) 47 (74.6)	< 0.001
Ascites, *n* (%) Absent Present	87 (67.4) 42 (32.6)	66 (100) 0 (0)	19 (30.2) 44 (69.8)	< 0.001
Esophago-gastric varices, *n* (%) No Yes	73 (56.6) 56 (43.4)	66 (100) 0 (0)	7 (11.1) 56 (88.9)	< 0.001
Splenomegaly, *n* (%) No Yes	76 (58.9) 53 (41.1)	47 (71.2) 19 (28.8)	29 (46.0) 34 (54.0)	0.004
Thrombocytopenia, *n* (%) No Yes	101 (78.3) 28 (21.7)	57 (86.4) 9 (13.6)	44 (69.8) 19 (30.2)	0.023
Clinical features of CEPH, *n* (%) 0 1 2 3	66 (51.2) 24 (18.6) 27 (20.9) 12 (9.3)	66 (100) 0 (0) 0 (0) 0 (0)	0 (0) 24 (38.1) 27 (42.9) 12 (19.0)	<0.001
Mean number of tumor nodules, (±STD)	2.1 ± 1.4	2.0 ± 1.5	2.3 ± 1.2	0.286
Number of tumor nodules, *n* (%) ≤3 >3	109 (84.5%) 20 (15.5%)	54 (81.8%) 12 (18.2%)	55 (87.3%) 8 (12.7%)	0.390
Tumor manifestation, *n* (%) Solitary Multifocal	58 (45.0) 71 (55.0)	35 (53.0) 31 (47.0)	23 (36.5) 40 (63.5)	0.059
Mean size largest nodule, cm, (±STD)	4.2 ± 2.3	4.1 ± 2.6	4.3 ± 2.0	0.665
Size largest nodule, *n* (%) ≤5 cm >5 cm	107 (82.9%) 22 (17.1%)	54 (81.8%) 12 (18.2%)	53 (84.1%) 10 (15.9%)	0.727
Mean total tumor diameter, cm, (±STD)	6.9 ± 3.8	6.5 ± 4.0	7.4 ± 3.6	0.180
Total tumor diameter, *n* (%) ≤10 cm >10 cm	111 (86.0) 18 (14.0)	56 (84.8) 10 (15.5)	55 (87.3) 8 (12.7)	0.688
Milan status, *n* (%) In Out	71 (55.0) 58 (45.0)	38 (57.6) 28 (42.4)	33 (52.4) 30 (47.6)	0.553
TACE prior LT, *n* (%) Yes No	94 (72.9) 35 (27.1)	49 (74.2) 17 (25.8)	45 (71.4) 18 (28.6)	0.719
Median AFP level, mg/dL, (range)	70 (2.7–46,926)	56 (2.7–5580)	100 (3.2–46,926)	0.151
Median CRP level, mg/dL, (range)	1 (0.1–9.5)	0.7 (0.1–4)	1 (0.2–9.5)	0.008
Median IL-6 level, ng/dL, (range)	17 (7–35)	15 (7–35)	17 (6–70)	0.001
Mean T_SUVmax_/L_SUVmax_, (±STD)	1.53 (0.79)	1.25 (0.45)	1.82 (0.94)	<0.001
PET status, *n* (%) Negative Positive	80 (62) 49 (38)	47 (71.2) 19 (28.8)	33 (52.4) 30 (47.6)	0.028
Grading, *n* (%) Well/moderate Poor	105 (81.4) 24 (18.6)	58 (87.9) 8 (12.1)	47 (74.6) 16 (25.4)	0.053
MVI, *n* (%) No Yes	79 (61.2) 50 (38.8)	46 (69.7) 20 (30.3)	33 (52.4) 30 (47.6)	0.044
Post-TACE tumor necrosis, *n* (%) >75% ≤75%	33 (35.1) 61 (64.9)	26 (53.1) 23 (46.9)	7 (15.6) 38 (84.4)	0.001

AFP, alfa-fetoprotein; CEPH, clinically evident portal hypertension; CRP, C-reactive protein; IL-6, interleukin-6; MELD, model of end-stage liver disease; MVI, microvascular invasion; T_SUVmax_/L_SUVmax_, tumor-to-normal liver standardized uptake value; PET, positron emission tomography; STD, standard deviation; TACE, transarterial chemoembolization.

**Table 2:** Uni- and multivariate predictors of post-LT HCC recurrence.

	Univariate		Multivariate	
Prognostic Factors	OR (95% CI)	*p* Value	OR (95% CI)	*p* Value
Male sex	1.14 (0.498–2.603)	0.758		
Age recipients’ > 60 years	1.48 (0.659–3.336)	0.341		
Virus-related liver cirrhosis	1.53 (0.617–3.778)	0.359		
Child–Pugh cirrhosis B or C	3.03 (1.196–7.693)	0.019		
MELD score > 20	3.00 (1.292–6.945)	0.011		
Ascites	4.62 (1.969–10.856)	0.001		
Esophago-gastric varices	4.60 (1.908–11.094)	0.001		
Splenomegaly	2.09 (0.923–4.738)	0.077		
Thrombocytopenia	2.17 (0.852–5.259)	0.106		
CEPH	5.19 (2.038–13.199)	0.001	6.88 (1.663–28.484)	0.008
Number of tumor nodules > 3	4.19 (1.546–11.362)	0.005		
Multifocal tumor manifestation	2.99 (1.222–7.342)	0.017		
Size of largest nodule > 5 cm	1.61 (0.590–4.412)	0.096		
Total tumor diameter > 10 cm	4.05 (1.437–11.390)	0.008		
Milan-out	4.21 (1.750–10.125)	0.001	3.90 (1.019–14.923)	0.047
No TACE prior LT	0.55 (0.240–1.279)	0.167		
AFP level > 100 mg/dL	8.87 (3.508–22.398)	<0.001	8.24 (2.122–31.962)	0.002
CRP level > 1 mg/dL	6.85 (2.82–16.612)	<0.001		
IL-6 level > 17 ng/dL	3.95 (1.672–9.344)	0.002	7.53 (1.863–30.394)	0.005
PET+ status	23.31 (7.366–73.813)	<0.001	26.38 (5.884–118.276)	<0.001

AFP, alfa-fetoprotein; CEPH, clinically evident portal hypertension; CI, confidence interval; CRP, C-reactive protein; IL-6, interleukin-6; LT, liver transplantation; MELD, model of end-stage liver disease; OR, odds ratio; PET, positron emission tomography; TACE, transarterial chemoembolization.

## Data Availability

Study data may be provided upon request by the corresponding author.

## Abbreviations

AFP	alfa-fetoprotein
CEPH	clinically evident portal hypertension
CNI	calcineurin inhibitor
CRP	C-reactive protein
CT	computed tomography
^18^F-FDG	^18^F-fluorodeoxy-glucose
HCC	hepatocellular carcinoma
HVPG	hepatic venous-portal gradient
IL-6	interleukin-6
LT	liver transplantation
MC	Milan criteria
MELD	model of end-stage liver disease
mTOR	mammalian target of rapamycin
MVI	microvascular invasion
OR	odds ratio
OS	overall survival
PET	positron emission tomography
PH	portal hypertension
PP	portal pressure
STD	standard deviation
TACE	transarterial chemoembolization
TME	tumor microenvironment
T_SUVmax_/L_SUVmax_	tumor-to-normal liver standardized uptake value
VEGF	vascular endothelial growth factor
